# Developing an Automated Gas Sampling Chamber for Measuring Variations in CO_2_ Exchange in a Maize Ecosystem at Night

**DOI:** 10.3390/s20216117

**Published:** 2020-10-27

**Authors:** Chaoqun Li, Wenting Han, Manman Peng, Mengfei Zhang

**Affiliations:** 1 College of Mechanical and Electronic Engineering, Northwest A&F University, Yangling 712100, China; chaoqun92@nwafu.edu.cn (C.L.); Tina0808@nwafu.edu.cn (M.P.); 2018050987@nwafu.edu.cn (M.Z.); 2 Key Laboratory of Agricultural Internet of Things, Ministry of Agriculture, Yangling 712100, China; 3 Institute of Soil and Water Conservation, Northwest A&F University, Yangling 712100, China

**Keywords:** dynamic closed chamber technology, static closed chamber technology, automated gas sampling chamber, CO_2_ exchange variation, environmental and physiological changes

## Abstract

The measurement of net ecosystem exchange (NEE) of field maize at a plot-sized scale is of great significance for assessing carbon emissions. Chamber methods remain the sole approach for measuring NEE at a plot-sized scale. However, traditional chamber methods are disadvantaged by their high labor intensity, significant resultant changes in microclimate, and significant impact on the physiology of crops. Therefore, an automated portable chamber with an air humidity control system to determinate the nighttime variation of NEE in field maize was developed. The chamber system can automatically open and close the chamber, and regularly collect gas in the chamber for laboratory analysis. Furthermore, a humidity control system was created to control the air humidity of the chamber. Chamber performance test results show that the maximum difference between the temperature and humidity outside and inside the chamber was 0.457 °C and 5.6%, respectively, during the NEE measuring period. Inside the chamber, the leaf temperature fluctuation range and the maximum relative change of the maize leaf respiration rate were −0.3 to 0.3 °C and 23.2015%, respectively. We verified a series of measurements of NEE using the dynamic and static closed chamber methods. The results show a good common point between the two measurement methods (N = 10, R^2^ = 0.986; and mean difference: ΔCO_2_ = 0.079${\;\mu \mathrm{mol}\;m}^{-2}s^{-1}$). This automated chamber was found to be useful for reducing the labor requirement and improving the time resolution of NEE monitoring. In the future, the relationship between the humidity control system and chamber volume can be studied to control the microclimate change more accurately.

## 1. Introduction

Accurate measurement of carbon dioxide exchange is of great significance for the development of crop growth models and the evaluation of crop greenhouse gas emissions. The canopy chamber method is more commonly used and is a single method for some small-scale experimental agriculture. At the crop canopy scale, it is also an important mode for the measurement of agricultural net ecosystem exchange (NEE) [1]. The canopy chamber method includes both closed and open chamber methods [2]. In the closed chamber method, the sample material is deployed in the enclosure of the closed chamber system, and the flux is estimated using the change in the CO_2_ concentration with time. In the closed chamber method, the air outside the chamber is continuously introduced into the chamber, and the CO_2_ flux is estimated based on the product of the air flow rate and the CO_2_ concentration difference in the air entering and leaving the chamber. The closed chamber method includes both dynamic and static closed chamber methods. 

In the field of agriculture and ecological environment, the dynamic closed chamber method is commonly used to measure NEE. In this method, the chamber is connected to an infrared gas analyzer (IRGA) through flexible inlet and outlet pipes, resulting in a closed circle structure with constant flow [3]. The use of the IRGA offers instant CO_2_ values that can be obtained directly in the field. Through a series of linear regression calculations, NEE is obtained, and the CO_2_ concentration value that changes with time in the chamber can be seen [4]. In previous research, it was noted that the CO_2_ flux can be calculated after continuously monitoring the concentration change in the chamber for 3–6 min [5,6,7]. To measure the daytime net CO_2_ exchange variation, eight to twelve [5] measurement cycles are implemented manually each day. Chamber measurement is labor intensive, which hinders night observation. To resolve this problem, Chen et al. developed an automated closed dynamic chamber system [8]. The chamber was equipped with an air cylinder device that could automatically open the chamber. However, one dynamic chamber was equipped with an IRGA, which meant that the utilization efficiency of the IRGA was very low. Liang, Inoue, and Fujinuma designed a fast response multi-chamber system for CO_2_ flux measurement on the soil surface through a series of studies [9]. A cover structure that allows automatic opening and closing is deployed inside the chamber, and 16 chambers are connected in parallel to a single IRGA using a 16-channel gas sampler designed in their research. This multi-chamber system structure can automatically monitor the CO_2_ flux. However, owing to the limitations of the gas tube, the distance at which the 16 chambers could be used was limited and the equipment was very large, meaning it was inconvenient to move.

The static closed chamber method is another commonly used method to measure NEE. In this method, after a certain period of time after the chamber is closed, a polypropylene syringe device with a three-way stopcock structure is adopted to realize manual gas sample collection [10]. Furthermore, gas chromatograph equipment is used to process and analyze gas samples (flame ionization detector and electron capture detector) to obtain the specific CO_2_ content [10,11,12]. Based on the gas sample’s CO_2_ concentration, NEE is obtained by calculation [13,14,15]. To measure the net CO_2_ exchange diurnal variation, manual gas acquisition is required at different times. To quantify the carbon dioxide exchange of multiple vegetations in coastal wetlands, Xi et al. collected gas every two or three hours during the night on each sampling day [16]. To obtain the change data of CO_2_ concentration and calculate NEE, the static closed chamber method must be closed for a long time (15–30 min), which generally causes significant temperature and humidity changes in the chamber and thus has a greater impact on crop physiology. Therefore, it is not appropriate to use the static chamber method in crops during the day. However, there is no light effect at night, which provides favorable conditions for developing an automated gas sampling static chamber for measurement of the CO_2_ exchange variation at night in cropland.

NEE of soil and crop ecosystems in different sites are quite different, even if these sites are in the same region. Therefore, when using the chamber method to measure NEE of an ecosystem, it is often necessary to conduct a large number of chamber experiments at different sites in the same region to obtain more accurate NEE values for that region. In a series of studies, Elsgaard et al. [17] analyzed the carbon dioxide flux data of eight regions in three geo-regions of Denmark. In their research, at each monitoring region, six sites were selected for measurement of their NEE using the dynamic closed chamber. Steven et al. [15] researched the relationship between biochar and CO_2_ emissions. In their research, 50 plots comprising a Latin rectangle were designed for a field experiment, and a static closed chamber was placed at each plot center to measure the CO_2_ flux. Therefore, it is necessary to design a low-cost, automated chamber to measure CO_2_ flux.

Crops can quickly sense changes in the environment, and the chamber can affect the environment and physiology of the crops during the measurement period [18]. It is necessary to research the effects of the chamber on the environment and physiology of the crops. These research objects mainly include the temperature, humidity, and the leaf temperature and respiration rate [1,4,18]. 

It can be seen from the previous research that both of the commonly used methods have high labor intensity, and the static closed chamber method has a significant impact on the micro-environment. Furthermore, a large number of chambers is often required to conduct the significant number of chamber experiments required to obtain more accurate NEE values for a region. These problems make it difficult to measure NEE at night. Thus, the current research aimed to: (1) design a low-cost and portable static closed chamber that can be automatically opened and closed, and collect gas in the chamber regularly for laboratory analysis; (2) design a humidity control system to prevent the rapid rise of humidity in the chamber, and determine the influence of the designed chamber on the micro-environment, leaf temperature and leaf respiration rate; and (3) validate the accuracy of the field maize NEE measured by the static chamber method.

## 2. Materials and Methods

### 2.1. Chamber Design and Operation

**Figure 1:**
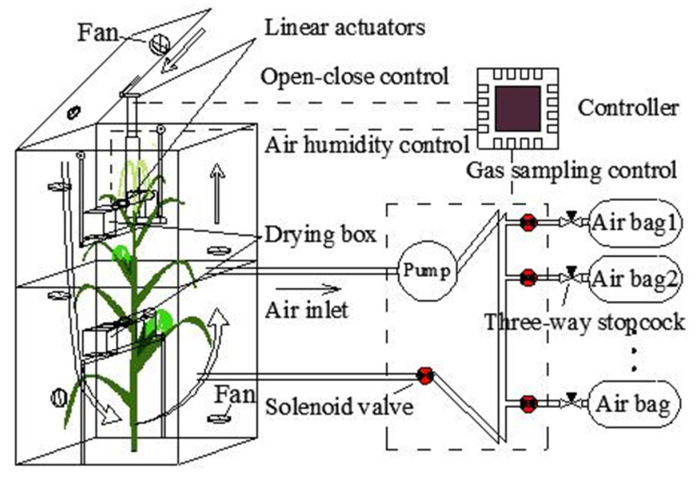
Schematic design of the static chamber system.

The static chamber system (Figure 1) includes three main components: an automated open–close chamber, a humidity control device, and a gas sampling device. Through the control system, the chamber can achieve the following three functions: (1) the chamber can be opened and closed for a certain period of time, and the cycle is continuous; (2) after the chamber is closed, the gas sampling device starts to work, collecting gas regularly; and (3) during the period that the chamber is closed, the humidity controller prevents the rapid rise of air humidity. The three main components of the system are described in detail in the following sections.

#### 2.1.1. Automated Open–Close Chamber

The main purpose of the chamber volume is to fit the size and phenotype (structure) of the maize. In this study the height, length, and width of the chamber are 110 × 54 × 54 cm, respectively. In the early vegetative stages, the height of the maize plants was short (less than 110 cm) and one chamber was sufficient to measure the maize NEE. However, after the late vegetative stage, the height of maize plants was taller. Therefore, two chambers were needed: one without a lid to cover the lower part of maize, and the other with a lid to cover the upper part (Figure 1). The chamber frame was made using a European standard 2020 aluminum extrusion profile. The frame was surrounded by transparent plexiglass (3 mm XT type 20070) and T-bolts, and butterfly wing nuts and screw glue fixes were used to fix them. Two adjacent plexiglass sheets were sealed with glue sticks using an electrically heated glue gun. The lid of the chamber was connected with a linear actuator, which could open and close the chamber regularly under the control of the controller. A self-adhesive rubber seal strip (Qingdao Yotile Rubber & Plastic Co., Ltd., Qingdao, China) was used on the chamber lid to ensure a tight fit. Fans were installed in the chamber, and the air flow direction formed by the fan is shown in Figure 1. The air flow rate adjacent to the chamber wall did not exceed 1.5 ms^−1^, which was significantly lower than the parameter at the center of the chamber (<0.8 ms^−1^). The wind speed was consistent with the wind speed in the chamber designed by Wei et al. and Matthias [5,6]. The air flow was used for mixing gas when the chamber was closed and for gas exchange with the external environment after the chamber was opened.

#### 2.1.2. Gas Sampling Equipment

The gas sampling device automatically collects the gas in the chamber using the pump (flow rate: 1.5 LPM) (model ZR320-01PM, Zhi Rong Co., Ltd., Dongguan, China) at 0, 5, 10, and 15 min after the chamber has been closed. There is a normal closed mini air solenoid valve (model AJK-F0509, Xiamen AJK Technology Co., Ltd., Fujian, China) in front of each aluminum foil gas sampling bag with a three-way stopcock (Dalian Hede Technology Co., Ltd., Dalian, China). The solenoid valve and the three-way stopcock are connected by a flexible tube. The gas tube of the gas sampling device consists of a branch tube (BT: tube between the air bag and the solenoid valve) and the main tube (MT: tube except BT; internal diameter: 3 mm, length: 120 cm). Before the experiment, the three-way stopcock switch is adjusted to ensure the BT is unblocked and each air bag is evacuated using a polypropylene syringe. The working process is detailed in Figure 1. First, the pump and the solenoid valve in the MT (SVMT) are turned on. Concurrently, the original gas in the MT is discharged and the MT is then filled with the gas in the chamber, within 4 s, using the gas pump. After completing this step, the SVMT is closed and the solenoid valve assembly in front of the air bag (SVAB) is then opened to start gas storage. After the gas collection is complete (about 100 mL, 5 s), the pump and SVAB are closed. After the first gas collection is complete, the gas collection device will collect gas at the next appointed time using the next air bag.

#### 2.1.3. Air Humidity Controller

CaCl_2_ is mainly used as a desiccant to achieve dehydration of natural gas [19]. While re-adsorbing water, it reaches a high-level hydration state, forming the corresponding CaCl_2_ salt solution. This process does not require the movement of parts or heating, and does not react with CO_2_ to a certain extent [19]. Thus, CaCl_2_ is widely used in the drying of natural gas and was used to control the air humidity in this study. Two humidity sensors were installed in the humidity control device, which were respectively installed inside and outside the chamber. When the difference between the internal humidity and the external humidity is more than 5%, the drying box (length, width, height: 15 × 15 × 10 cm) for CaCl_2_ is opened by a linear actuator. When the difference is less than 3%, or if the chamber is in the open state, the drying box is closed again by the linear actuator. The drying box has two layers: the upper layer contains the CaCl_2_ (height: 4 cm) and the lower layer is used to collect the water. 

### 2.2. Temperature and Humidity in the Chamber Test

The humidity and temperature of the air have a significant influence on the physiological state of crops. Therefore, sensors are used to measure air temperature and humidity (model HOBO U23-001, Onset Computer Corp, Bourne, MA, USA) inside and outside the chamber, and placed a row away from the static chamber. The temperature and humidity were measured twice, and the duration of each measurement was the entire night. In the first measurement (date: 1/7/2019) a 110 cm high chamber was used to cover the maize in the vegetative stages, and in the second measurement (date: 21/7/2019) two 110 cm high chambers were used for the taller maize plants in the reproductive stages: one without a lid was used to cover the lower part of the maize, the other chamber with a lid was used to cover the upper part. The sensors were placed in the center of the chamber and the values were logged every 10 s.

### 2.3. Leaf Temperature Test

**Figure 2:**
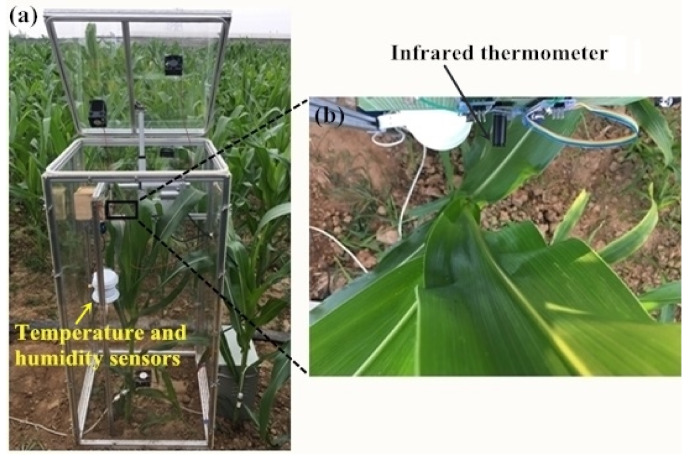
Photos of leaf temperature, air humidity, and temperature sensors in the chamber: (**a**) location of the sensors within the chamber and (**b**) location of the leaf temperature sensor.

The leaf temperature inside the chamber was measured twice and the duration of each measurement was the entire night. In the first measurement (date: 3/7/2019), a 110 cm high chamber was used to cover the maize in the vegetative stages. In the second measurement (date: 24/7/2019) two 110cm high chambers were used for the taller maize plants in the reproductive stages: one without a lid was used to cover the lower part of the maize, the other chamber with a lid was used to cover the upper part. An infrared thermometer (IRT) (model MLX90614-DCI, Melexis Technologies NV, Iber, Belgium) with an accuracy of 0.2 °C and a measurement resolution of 0.02 °C provided non-contact measurement of the leaf temperature. The IRT was positioned approximately 12 cm from the leaf (field of view = 10 mm diameter) (Figure 2b) and the leaf temperatures were logged every 10 s.

### 2.4. Leaf Respiration Rate Test

**Figure 3:**
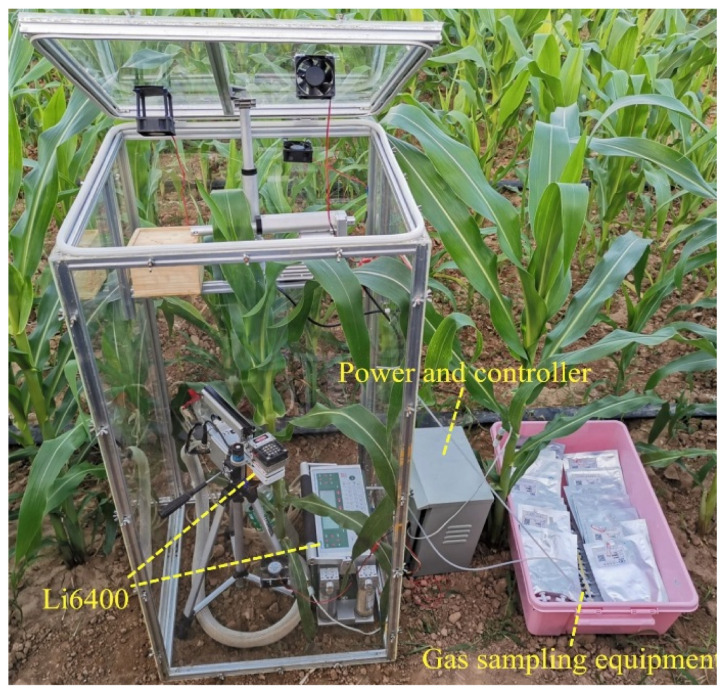
Experimental configuration used to measure the respiratory rate of the leaf using the Li6400 and net ecosystem exchange (NEE) of the maize field was measured by the developed static closed chamber.

The Li 6400 portable photosynthesis system (Li-Cor Inc., Lincoln, NE, USA) can effectively measure the leaf respiration inside the chamber (Figure 3). The leaf respiration experiment was performed four times. Experiments 1 and 2 (4/7/2019) were performed during the vegetative stages and a 110 cm high chamber was used to cover the entire plant. Experiments 3 and 4 (26/7/2019) were performed during the reproductive stages and two 110 cm chambers were used to measure the taller plants: one without a lid was used to cover the lower part of the maize, the other chamber with a lid was used to cover the upper part. Each experiment lasted 18 min and the first 3 min were performed under ambient air; the remainder were performed under the micro-environment of the chamber by covering the maize and the Li6400 using the chamber with closed lid. The respiration rate was automatically recorded every 1 min. 

### 2.5. Gas-Exchange Measurements

This chamber can be used as a dynamic closed chamber or a static closed chamber. Compared with static closed chamber method, the dynamic closed chamber method requires a shorter time to complete a NEE measurement and the environment in the dynamic chamber changes less. Dynamic closed chambers are generally considered to be more accurate than those used in static methods [20]. Furthermore, because the duration of each experiment is only 15 min, there was little variation in maize field NEE in one plot and we assume that the NEE value of maize in this period is a constant value. Therefore, in this study, we used the dynamic chamber method to evaluate the measurement accuracy of the static chamber method. For the static chamber method, the gas sampling device (Figure 3) collects the gas in the chamber using the pump at 0, 5, 10, and 15 min after the chamber was closed. Then, the CO_2_ concentration of the samples was analyzed using gas chromatography (GC-2010PLUS, Shimadzu, Japan) within 24 h. For the dynamic chamber method, the variation in the CO_2_ concentration was monitored using the Li6400 for approximately 5 min and the data were logged every 10 s. In each measurement, the two methods were carried out at the same time using the same chamber. The experiments were performed about every six days, at around 10:00 PM each time. The NEE was determined using the following equation [15]:

$$F_{{\mathrm{CO}}_{2}}=k_{{\mathrm{CO}}_{2}}\frac{273}{T}\frac{V}{A}\frac{\Delta c}{\Delta t}$$

where $k_{{\mathrm{CO}}_{2}}$ is the gas constant at 273.15 K, 101.325 KPa (0.536 g C L^−1^), ${\;F}_{{\mathrm{CO}}_{2}}$ is the flux of CO_2_, T is the air temperature in the chamber during the measurement period, A is the surface area of the collar of the chamber, V is the volume of the chamber, and $\frac{\Delta c}{\Delta t}$ is the change in the CO_2_ concentration.

## 3. Results and Discussion

### 3.1. Air Temperature and Humidity Changes Inside and Outside the Chamber

**Figure 4:**
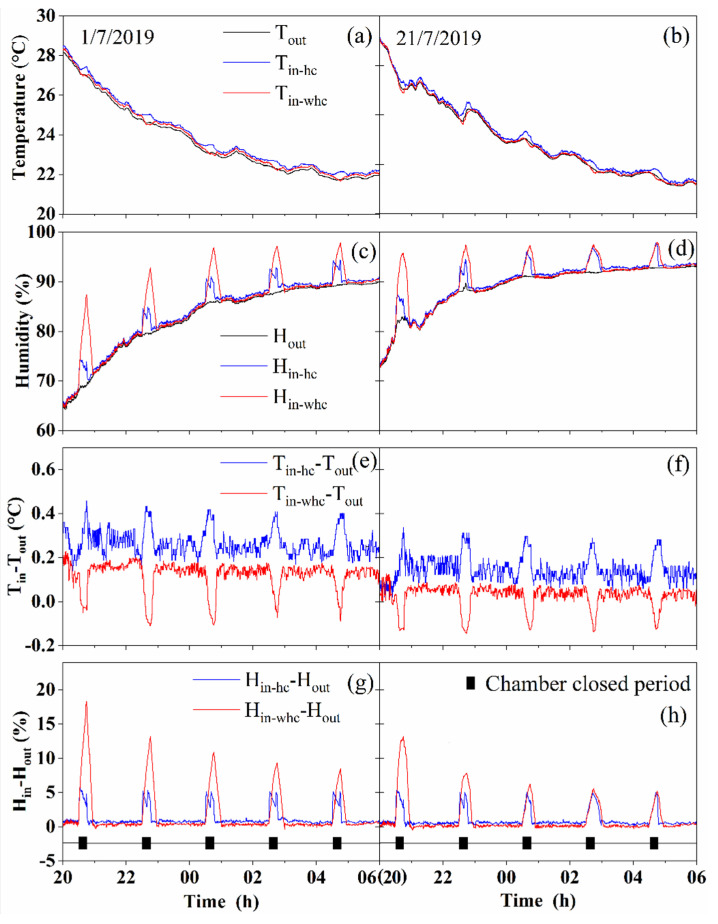
The temperature and humidity variations of the ambient temperature and humidity inside the chamber using the humidity controller and inside the chamber without the humidity controller. On 1/7/2019, one 110 cm high chamber was used to cover the maize plants in the vegetative stages. On 21/7/2019, two 110 cm high chambers were used for the taller maize plants in the reproductive stages. (**a**,**b**): Air temperature variation outside the chamber (T_out_), inside the chamber with the humidity controller (T_in-hc_) and inside the chamber without the humidity controller (T_in-whc_). (**c**,**d**): Air humidity variation outside the chamber(H_out_), inside the chamber with the humidity controller (H_in-hc_) and inside the chamber without the humidity controller (H_in-whc_). (**e**,**f**): The variation of T_in-hc_ − T_out_ and T_in-whc_ − T_out_. (**g**,**h**): The variation of H_in-hc_ − H_out_ and H_in-whc_ − H_out_.

The temperature and humidity results are shown in Figure 4. During the measurement day, the variation in the air temperature inside the chamber with the humidity controller (T_in-hc_), the variation in the air temperature inside the chamber without the humidity controller (T_in-whc_), and the variation in the air temperature outside the chamber (T_out_) showed a downward trend from 8 PM to 6 AM (Figure 4a,b). When the chamber was in the open state, T_in-hc_ − T_out_ and T_in-whc_ − T_out_ (Figure 4e,f) fluctuated around a constant value. When the chamber was in the closed state, T_in-hc_ − T_out_ revealed an upward trend and the maximum difference from several experiments was 0.457 °C. T_in-whc_ − T_out_ showed a downward trend and the maximum difference from several experiments was 0.148 °C. When the chamber changed from a closed state to an open state, T_in-hc_ − T_out_ rapidly decreased to the pre-closure levels and T_in-whc_ − T_out_ rapidly increased to the pre-closure levels.

During the measurement day, humidity variation inside the chamber using the humidity controller (H_in-hc_), humidity variation inside the chamber without the humidity controller (H_in-whc_), and air humidity variation outside (H_out_) the chamber showed an upward trend from 8 PM to 6 AM (Figure 4c,d). When the chamber was in the open state, similar to the temperature results, H_in-hc_ − H_out_ and H_in-whc_ − H_out_ (Figure 4g,h) also fluctuated around a constant value. When the chamber was in the closed state, H_in-hc_ − H_out_ increased slightly, with a maximum of 5.6% from several experiments. H_in-whc_ − H_out_ revealed a rapid rising trend in several experiments, and the maximum value was 18.3%. When the chamber changed from the closed state to the open state, H_in-hc_ − H_out_ and H_in-whc_ − H_out_ rapidly decreased to the pre-closure levels.

Many studies concerning temperature and humidity variation in the dynamic chamber method have been published. Steduto et al. [1] found that the temperature variation is basically in the range of 1–2 °C in the CO_2_ exchange measurement period. If there is no climate regulation, it is more difficult to maintain the temperature change in the closed chamber in the range of 2 °C [1]. Through research, Gabriele developed a dynamic closed chamber that can effectively monitor the CO_2_ exchange dynamics of bushes [21]. The coverage area of the chamber is 0.64 m^2^ (length and width are 0.8 × 0.8 m). To be suitable for different height bushes, two or more bases were designed and the chamber can be adjusted from 0.6 to 1.3 meters by stacking the bases. In his study, the temperature variations were always below 1.3 °C in the measurement period. The recorded temperature change value is similar to that of the larger volume chamber [7,22,23] and is much lower than the 2 °C recorded in many other closed chambers [1,24]. 

For the chamber method, the increase of the air humidity in the chamber is a general problem. Droesler [6] used the dynamic closed chamber method to measure NEE of bog and their research showed that the maximum change of humidity during the measurement period reaches 20%. The relative humidity increase in the chamber designed by Gabriele [21] ranged between 4 and 20% in the CO_2_ exchange measurement period, and the average value of humidity increase was 13%. Due to the use of the humidity control system, the maximum humidity change in the chamber developed in this study was 5.6% in the experiments.

### 3.2. Leaf Temperature Variation Inside the Chamber

**Figure 5:**
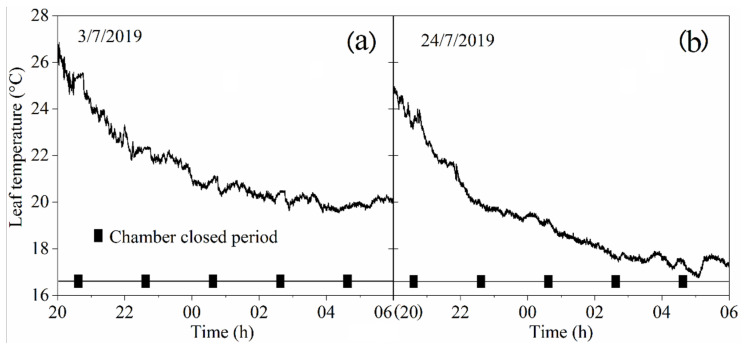
Leaf temperature change measured using two different height chambers: (**a**) on 3/7/2019, one 110 cm height chamber was used to cover a maize plant in the vegetative stages; and (**b**) on 24/7/2019, two 110 cm high chambers were used to cover a taller maize plant in the reproductive stages, as shown in Figure 1.

The leaf temperature variation results are shown in Figure 5. The temperature of the leaf inside the chamber showed a downward trend from 8 PM to 6 AM. When the chamber was in the closed state, the maize leaf temperature changed slightly and the percentage distribution of the temperature variation ranges (ΔT) during the closed chamber period can be seen in the Table 1. The ΔT was mainly distributed between −0.3 and 0.3 °C when the chamber was in the closed state. The values of ΔT_max_ and ΔT_min_ were 0.37 and −0.38 °C, respectively.

Use of the chamber will lead to a rise in the leaf temperature during daytime measurements. McPherson et al. measured the maize leaf temperature inside the chamber after the chamber was closed. Results show that the average value of the leaf temperature increases by 1.3 °C within 8 s and the average value of the leaf temperature increases by 2.2 °C within 25 s [25]. Research by Reicosky and Wagner found that the temperature of corn leaves increased from 2 to 4°C within 60 s after the chamber was closed and the leaf temperatures recovered to their approximate pre-closure temperature within 60 s after the chamber was opened [18]. 

Luo developed a portable closed chamber with a footprint area of 1.5 m^2^ that mainly realizes the evapotranspiration measurement of soybean and corn [26]. To match the growth stages of various crops, Luo designed chambers with three different heights (0.6, 1.0, and 1.6 m) and in each measurement stage, the temperature difference is maintained at 2 °C before and during the CO_2_ exchange measurement.

### 3.3. Response of Maize Leaf Respiration Rate to the Chamber

**Figure 6:**
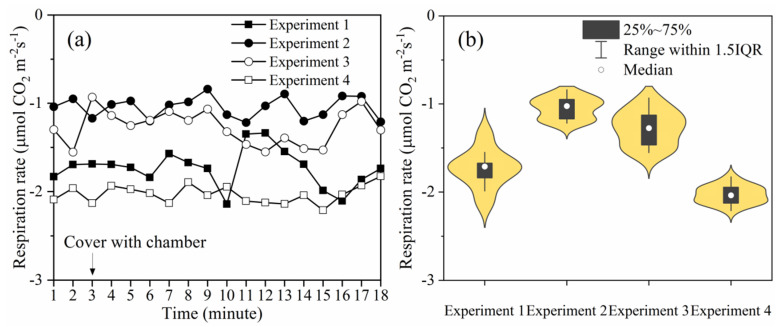
In experiments 1 and 2, a single 110 cm height chamber was used to cover the maize in the vegetative stages. In experiments 3 and 4, two 110 cm high chambers were used to cover the taller maize plants in the reproductive stages, as shown in Figure 1. (**a**) Graph showing the changes in the respiration of leaves before and after the maize was covered. (**b**) Graph showing the violin plot with a box plot for each experiment (IQR = interquartile range).

The variation in the leaf respiration rate after the chamber was closed is shown in Figure 6a. The leaf respiration rate fluctuates around the value observed before the maize was covered by the chamber. The average value for the respiration rate of maize in each experiment was between −1.05319 and −2.06109, and the maximum relative change in the respiration rate was 23.2% after the maize was covered by the chamber. 

The distribution of the data from the four experiments is represented using a violin plot with a box plot (Figure 6b), in which the width of each violin chart is the data density of the maize leaf respiratory rate value. In the content of Figure 6b, through analysis and research, it was found that the data was mainly distributed close to the average value obtained for the respiratory rate before the chamber was covered. The percentage distributions of the relative change of leaf respiration rate are reported in Table 2. Sixty percent of the data was distributed between −10 and 10. The proportion of ΔR ≤ −20 was 20% and the proportion of ΔR ≥ 20 was also 20%. The data of leaf respiration rate measured in the natural environment (three minutes before the maize and Li6400 were covered by the chamber) were statistically analyzed. As with the measurement in the chamber, ΔR ≤ −20 and ΔR ≥ 20 also existed when measuring the leaf respiration rate in the natural environment. Therefore, there was no significant increase or decrease in leaf respiration rate during the period when the chamber was closed.

### 3.4. Comparison of the CO_2_ Exchange Measurements Obtained Using Static and Dynamic Closed Technology

**Figure 7:**
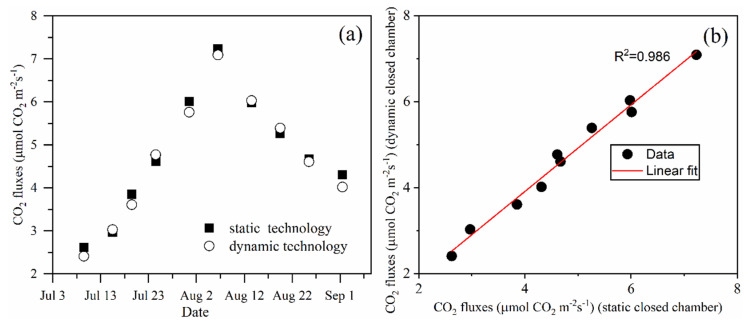
(**a**) Graph showing the maize field CO_2_ flux measured using static and dynamic technology. (**b**) Graph showing the linear regression analysis of the CO_2_ flux data measured using static and dynamic technology.

Figure 7a shows the CO_2_ exchange measurements obtained using static and dynamic closed chamber technology at 10 PM on different days. The CO_2_ exchange values obtained using static and dynamic closed technology were in very good agreement throughout the duration of the measurements. The difference in NEE measured using dynamic and static methods was between −0.29 and 0.16, and the relative errors were distributed between 8.71% and −3.35%. The minimum value was observed on the July 3, which may be because the biomass of the maize was relatively small and the CO_2_ flux increased continuously from July 3 to August 7. The CO_2_ flux then began to decrease as the date increased.

The values measured using the two methods on the same day were used to obtain the coordinate point (Figure 7b). There is a significant correlation between the CO_2_ flux measurements obtained using the static and dynamic closed chamber methods. This correlation yields an R^2^ of 0.986. The difference throughout the measurement data has a mean difference of 0.079 ${\mu \mathrm{mol}\;m}^{-2\;}s^{-1}$ between the static and dynamic closed technology.

The open chamber system can be used for long-term CO_2_ flux monitoring and research, but the portability is poor and the operation is not simple. Thus, the open chamber system is only used for CO_2_ flux measurement of a small number of canopy patches. The closed chamber system is more suitable for rapid measurement; a single portable chamber can be used on a variety of patches, but this method has a great impact on the micro-environment and requires significant labor input to measure the change of CO_2_ flux. The automated gas sampling chamber developed in this study allows unattended night measurements of CO_2_ exchange in a maize ecosystem, and results in relatively little change of the micro-environment inside the chamber.

However, the developed chamber in this study also has some limitations. Higher soil moisture will cause the air humidity to increase rapidly, which may exceed the ability of the humidity controller to reduce air humidity in the chamber. The response of humidity in the chamber to desiccant content and chamber size needs to be studied in the future. The method of measuring soil CO_2_ flux is the same as that used to measure the maize ecosystem CO_2_ flux. The developed chamber method can be used to measure the soil emissions by changing the chamber size. However, due to the difference of soil moisture, crop root, and microbial population, the soil flux in different sites is quite different, even if these sites are in the same region. Therefore, when using the developed chamber method to measure the CO_2_ flux of ecosystem, it is often necessary to conduct several chamber experiments at different sites in the same region to obtain more accurate CO_2_ flux measurements in the region.

## 4. Conclusions

The developed chamber can automatically open and close, and regularly collect gas for laboratory analysis, thus reducing the required labor input and improving the time resolution of NEE monitoring. 

A humidity control system was developed to reduce the influence of the chamber on the microclimate and crop physiology. Due to the application of a humidity control system, the maximum difference between outdoor and indoor humidity and temperature was 5.6% and 0.457 °C, respectively. The range for the changes in leaf temperature and the maximum relative changes of maize leaf respiration rate were −0.3 to 0.3 °C and 23.2015%, respectively, during the NEE measurement period. The effects of the chamber on the environment and physiology of crops were weak.

The automated chamber presented in this work allows measurement of CO_2_ exchange in the maize ecosystem at night. A comparison of the dynamic chamber method and the method used in this study showed that the two methods have a good consistency.

It is a challenge to manually perform NEE measurement experiments at night. The chamber method designed in this study provides an alternative for solving such problems. Furthermore, the low cost of the chamber means it is easy to deploy in situations in which a larger number of chamber is required to measure NEE.

## Figures and Tables

**Table 1:** Percentage distribution of the leaf temperature variation ranges during the closed chamber period.

Day	ΔT≤−0.3 (%)	−0.3<ΔT≤0 (%)	0 <ΔT<0.3 (%)	ΔT≥0.3 (%)	ΔT_max_ (°C)	ΔT_min_ (°C)
3 July 2019	0	10.8	89.2	0	0.36	−0.12
24 July 2019	3.1	54.9	39.7	2.3	0.37	−0.38

**Table 2:** Percentage distribution of the relative change of leaf respiration rate.

ΔR≤−20 (%)	−20<ΔR≤−10 (%)	−10<ΔR<0 (%)	0<ΔR<10 (%)	10≤ΔR<20 (%)	ΔR≥20 (%)
6.67	13.33	38.33	21.67	13.33	6.67
